# Resveratrol and pterostilbene epigenetically restore PTEN expression by targeting oncomiRs of the miR-17 family in prostate cancer

**DOI:** 10.18632/oncotarget.4877

**Published:** 2015-08-06

**Authors:** Swati Dhar, Avinash Kumar, Agnes M. Rimando, Xu Zhang, Anait S. Levenson

**Affiliations:** ^1^ Cancer Institute, University of Mississippi Medical Center, Jackson, Mississippi, USA; ^2^ United States Department of Agriculture, Agricultural Research Service, Natural Products Utilization Research Unit, University, Mississippi, USA; ^3^ Center of Biostatistics and Bioinformatics, University of Mississippi Medical Center, Jackson, Mississippi, USA; ^4^ Department of Pathology, University of Mississippi Medical Center, Jackson, Mississippi, USA

**Keywords:** oncomiRs, prostate cancer epigenetics, PTEN, pterostilbene, resveratrol

## Abstract

In recent years, not only has the role of miRNAs in cancer become increasingly clear but also their utilization as potential biomarkers and therapeutic targets has gained ground. Although the importance of dietary stilbenes such as resveratrol and pterostilbene as anti-cancer agents is well recognized, our understanding of their miRNA-targeting capabilities is still limited. In our previous study, we reported that resveratrol downregulates PTEN-targeting members of the oncogenic miR-17 family, which are overexpressed in prostate cancer. This study investigates the resveratrol and pterostilbene induced miRNA-mediated regulation of PTEN in prostate cancer. Here, we show that both compounds decrease the levels of endogenous as well as exogenously expressed miR-17, miR-20a and miR-106b thereby upregulating their target PTEN. Using functional luciferase reporter assays, we demonstrate that ectopically expressed miR-17, miR-20a and miR-106b directly target PTEN 3′UTR to reduce its expression, an effect rescued upon treatment with resveratrol and pterostilbene. Moreover, while stable lentiviral expression of miR-17/106a significantly decreased PTEN mRNA and protein levels and conferred survival advantage to the cells, resveratrol and more so pterostilbene was able to dramatically suppress these effects. Further, pterostilbene through downregulation of miR-17-5p and miR-106a-5p expression both in tumors and systemic circulation, rescued PTEN mRNA and protein levels leading to reduced tumor growth *in vivo*. Our findings implicate dietary stilbenes as an attractive miRNA-mediated chemopreventive and therapeutic strategy, and circulating miRNAs as potential chemopreventive and predictive biomarkers for clinical development in prostate cancer.

## INTRODUCTION

A growing body of experimental evidence suggest an aberrant expression of microRNAs (miRNAs, miRs) in cancer, which can act as oncogenes or tumor suppressors defining the fate of tumor formation [1–3]. Since their discovery as short, single-stranded, noncoding RNA molecules that act as posttranscriptional regulators of gene expression [4, 5], our understanding of the mechanisms of miRNA action has considerably increased. In general, miRNAs bind to sequences in the 3′UTR (3′ untranslated region) of target genes and decrease the stability of nascent mRNA or/and protein translation, which results in decreased production of the target protein [6].

Most importantly, miRNAs have a number of desirable characteristics for clinical application: disease specificity, exceptional stability in various types of clinical samples, and ability to respond to therapy [7]. These features together with presence of circulating miRNAs in cell-free fraction of blood, i.e. serum and plasma, make miRNAs incomparable potential biomarkers for cancer diagnosis, prediction, and prognosis, which in turn, mark them as valuable targets for the clinical development of anticancer agents [7–9].

In prostate cancer, changes in expression of miRNAs are associated with clinicopathological parameters such as Gleason score and recurrence [8, 10–12]. In particular, there is an overexpression and amplification of oncogenic miR-17∼92 and miR-106b∼25 clusters in prostate cancer [10, 13, 14]. We too, using miRNA profiling in LNCaP cells, reported significant expression of oncogenic miR-17∼92, miR-106a∼363, and miR-106b∼25 clusters, some members of which target the tumor suppressor gene PTEN (Phosphatase and Tensin homolog) [15]. PTEN is frequently defective in prostate cancer as its deletions/mutations are found in primary and metastatic disease [16]. It was speculated that *PTEN* heterozygosity, when accompanied by miRNA-mediated down-regulation, might be more effective at promoting tumorigenesis than complete homozygous loss of *PTEN* [17]. Since homozygous *PTEN* deletion is not common in human prostate cancer, the role of epigenetic regulators, such as miRNAs, becomes more important in contributing to PTEN expression and activity. This, in turn, opens the door for potential epigenetic therapies, including dietary compounds with the ability to modulate PTEN abundance by suppressing oncogenic miRNAs.

Our knowledge of anticancer therapies, especially dietary compounds that can control aberrant miRNA expression is relatively limited [18, 19]. Dietary stilbenes, such as resveratrol (Res) (*trans*-3, 5, 4′-trihydroxystilbene) and its potent natural analog pterostilbene (Pter) (*trans*-3, 5-dimethoxy-4′-hydroxystilbene), are known for their antioxidant, anti-inflammatory, cardioprotective and anticancer activities [20–22]. Both compounds have pleiotropic anticancer activities that include induction of apoptosis and inhibition of angiogenesis and metastasis [23–31]. Since miRNAs regulate all aspects of cancer biology, one mechanism of anticancer effects of dietary stilbenes may as well be through modulation of miRNAs.

We previously showed that resveratrol significantly altered miRNA profiles in prostate cancer, including members of the oncogenic miR-17 family, predicted to target PTEN [15]. Upregulation of PTEN protein levels by resveratrol in prostate cancer have been reported earlier [15, 32]. One mechanism of PTEN upregulation by resveratrol, reported by us recently, is inhibition of metastasis-associated protein 1 (MTA1)-mediated deacetylation and inactivation of PTEN [27]. However, the role of dietary stilbenes such as resveratrol and pterostilbene in modulating miRNA-mediated regulation of PTEN in prostate cancer has not been investigated.

In the current study, we hypothesized an oncomiR-mediated inhibition of PTEN and provided experimental validation on the ability of resveratrol and pterostilbene to rescue this effect. Our results indicate that both resveratrol and pterostilbene exert their anticancer effects, at least in part, via repression of several members of the oncogenic miR-17 family. We show that by inhibiting these oncomiRs, resveratrol and pterostilbene rescue the expression of PTEN tumor suppressor. This is the first report on dietary stilbenes’ miRNA-mediated regulation of PTEN in prostate cancer.

## RESULTS

### Resveratrol rescues miR-mediated downregulation of PTEN in prostate cancer cells

**Figure 1:**
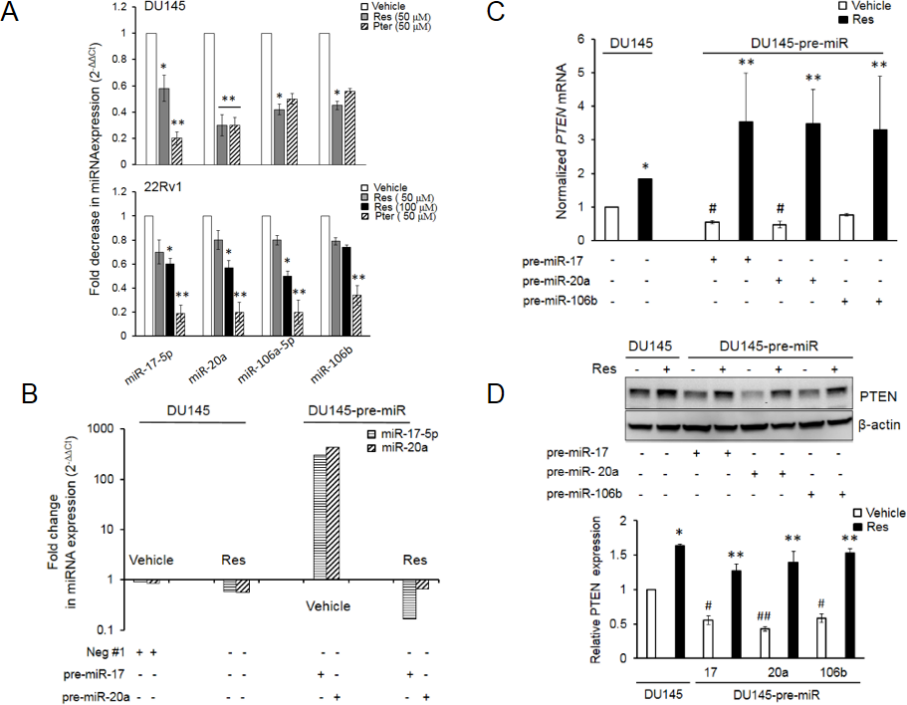
Resveratrol and pterostilbene reversal of miR-mediated downregulation of PTEN mRNA and protein **A.** Resveratrol and pterostilbene induced downregulation of miR-17 family oncomiRs in DU145 and 22Rv1 PCa cells. Cells were treated with resveratrol and pterostilbene at indicated concentrations, miRNA was isolated and real time PCR was performed as detailed in Materials and Methods. All samples were within threshold cycle (C_t_ < 35). **B.** Resveratrol directly targets ectopically expressed miRs in DU145 cells. Relative abundance of miR-17-5p and miR-20a was measured by real time PCR. **C, D.** Resveratrol significantly restored and enhanced PTEN mRNA (C) and protein (D) expression in the presence of ectopically overexpressed miRs, which diminished PTEN expression in vehicle-treated cells. Fold changes in expression of miRNAs and mRNA was calculated by the 2^−ΔΔCt^ method. Data represent the mean ± SEM from three independent experiments. Quantitation of blots was performed using Image J software. Comparisons between non- transfected and miR-transfected samples (#) and vehicle-treated and compound-treated samples (*) are depicted. #*p* < 0.05; ##*p* < 0.01; *p < 0.05; **p < 0.01. Res, resveratrol; Pter, pterostilbene.

Previous report from our laboratory showed that the expression levels of miRs from oncogenic miR-17∼92, miR-106a∼363 and miR-106b∼25 clusters were downregulated in LNCaP and DU145 PCa cells treated with 50 μM resveratrol [15]. Based on their seed sequences, the miRNAs of these clusters are grouped into four families, one of which is the miR-17 family that consists of miR-17, miR-20a and b, miR-106a and b and miR-93 [33]. According to miRanda/MicroCosm [34], TargetScan [35] and Diana-microT-CDS [36] prediction algorithms, miRs-17, -20a and -106a and b target tumor suppressor PTEN gene. Independently, we observed an upregulation of PTEN protein levels in DU145 and 22Rv1 cells [15, 27] leading to downregulation of PI3K-Akt signaling by resveratrol [27] and hypothesized that this effect could be regulated, at least in part, by resveratrol-modulation of PTEN-targeting miRNAs. Quantitative RT-PCR analysis confirmed that both resveratrol and its natural analog pterostilbene significantly downregulated miRs-17, -20a, -106a and -106b in DU145 and 22Rv1 prostate cancer cells that express wild type PTEN (Figure 1A).

To establish a direct effect of resveratrol on miRNAs, we examined whether resveratrol regulates ectopically expressed miRs in prostate cancer cells. Resveratrol treatment decreased ectopic miRNA expression similar to its effect on endogenous miRNA (Figure 1B). Further, ectopic overexpression of miRs -17 or -20a or -106b alone resulted in downregulation of their target PTEN mRNA and protein in DU145 cells, which was rescued by treatment with 50 μM of resveratrol (Figure 1C and 1D). Cumulatively, these results suggest that resveratrol's rescue of PTEN mRNA and protein is mediated, at least in part, through its ability to downregulate oncomiRs of the miR-17 family involved in targeting PTEN.

### Resveratrol and pterostilbene reverse targeting of PTEN 3′UTR by oncomiRs

**Figure 2:**
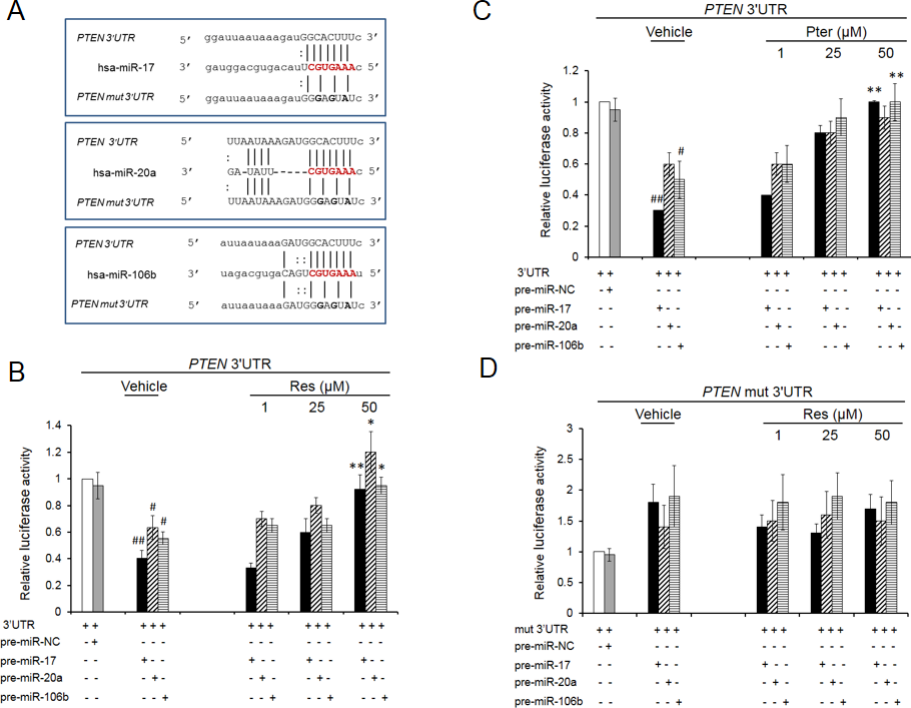
Resveratrol and pterostilbene rescue PTEN inhibition by oncomiRs -17, -20a and -106b **A.** Schematic representation of the predicted target sites of miRs-17, -20a and -106b in the 3′UTR of PTEN mRNA. The miRNA seed sequence is shared by all three miRs and shown in red. Mutated nucleotides in the 3′UTR are shown in bold. **B, C.** Resveratrol and pterostilbene oppose *PTEN* 3′UTR targeting. Relative luciferase activity in DU145 cells co-transfected with wt *PTEN* 3′UTR along with either pre-miR-17, -20a or 106b and treated with resveratrol (B) or pterostrilbene (C) **D.** Co-transfections with mutated 3′UTR did not show any inhibitory effect on luciferase activity. MiR-negative#1 was used as a negative control (miR-NC). Values are normalized to Renilla luciferase activity and relative to 3′UTR/EV (Empty Vector) ratio which is set at 1. Data represent the mean ± SEM from four independent experiments. Comparisons between non-transfected and miR transfected samples (#) and vehicle and compound-treated samples (*) are shown. #*p* < 0.05; ##*p* < 0.01; *p < 0.05; **p < 0.01. Res, resveratrol; Pter, pterostilbene.

To validate whether PTEN is a direct target gene of miRs-17, -20a and -106b, we employed the dual-luciferase reporter assay. For this, PTEN 3′UTR sequence (535 bp) (Supplementary Figure S1) containing either the wild type seed match for all three miRs (GCACTTT) or its mutant (mut) form (GGAGTAT) was amplified and cloned into the pMIRGLO vector, downstream of the luciferase reporter gene. The overall target specificity for miR-17, miR-20a and miR-106b defined by miRanda (http://www.microrna.org) was comparable for these three miRs (Figure 2A). The data showed that the co-expression of miR-17 or miR-20a or miR-106b with wild type 3′UTR (Figure 2B and 2C) but not with mutant 3′UTR (Figure 2D) significantly suppressed the luciferase activity, indicating that these miRs directly target the 3′UTR of PTEN. When cells were treated with either resveratrol or pterostilbene, there was a reversal of miRs’ inhibitory effects with wild type 3′UTR (Figure 2B and 2C) and no changes with mutant 3′UTR (Figure 2D). These results indicated that stilbenes can reverse the targeting of PTEN 3′UTR by these miRNAs.

### Resveratrol and pterostilbene restore PTEN mRNA and protein expression in DU145 cells stably overexpressing miR-17/106a

**Figure 3:**
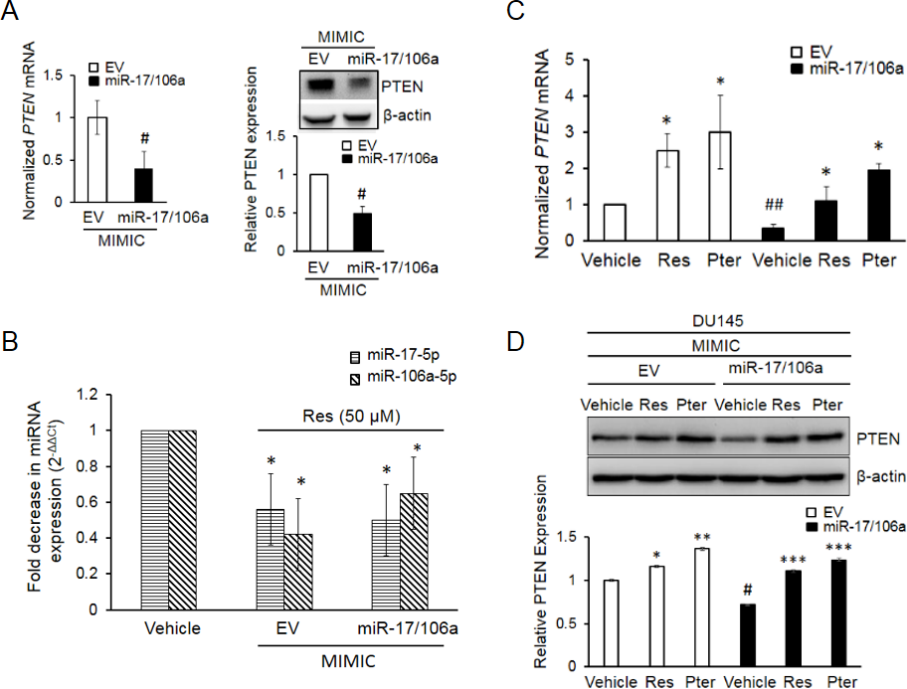
Establishment and characterization of DU145 cells stably overexpressing miR-17/106a **A.** PTEN mRNA (left) and protein (right) were significantly decreased in miR-17/106a MIMIC compared to EV MIMIC cells. PTEN mRNA expression was detected by real time PCR and protein was detected by western blot. **B.** Resveratrol inhibits relative abundance of miRs-17 and -106a in EV and miR-17/106a MIMIC cells as detected by real time PCR. **C, D.** Resveratrol and pterostilbene enhanced expression of PTEN mRNA (C) and protein (D) in EV MIMIC and miR-17/106a MIMIC cells. Fold change in expression of miRNAs and mRNA was calculated by the 2^−ΔΔCt^ method. Data represent the mean ± SEM from at least three independent experiments. Quantitation of blots was performed using Image J software. Comparisons between non-transfected and miR transfected samples (#) and vehicle and compound-treated samples (*) are shown. #*p* < 0.05; ##*p* < 0.01; *p < 0.05; **p < 0.01; ***p < 0.001. EV, Empty vector, Ctrl, Control, Res, resveratrol; Pter, pterostilbene.

In order to further explore the functional relevance of PTEN-targeting miRNAs, we established DU145-Luc cells stably overexpressing miR-17/106a referred here after as miR-17/106a MIMIC (Supplementary Figure S2A and S2B). Stable overexpression of miR-17/106a caused a significant downregulation of PTEN mRNA and protein levels (Figure 3A). Moreover, resveratrol efficiently downregulated the levels of ectopically expressed miRs (Figure 3B) resulting in rescue of PTEN mRNA (Figure 3C) and protein (Figure 3D). As seen in Figures 3C and 3D, pterostilbene demonstrated marginally stronger effect in restoring both PTEN mRNA and protein levels. In addition, we observed that overexpression of miRs confers survival advantage to these cells, and the inhibitory effects of both agents on cell growth were more pronounced in miR-17/106a MIMIC cells compared to empty vector (EV) control (Supplementary Figure S2C). These results suggested that the stilbenes rescue PTEN expression by downregulation of miR-17-5p and miR-106a-5p.

### Pterostilbene effectively diminishes tumor growth in miR-17/106a overexpressing xenografts

**Figure 4:**
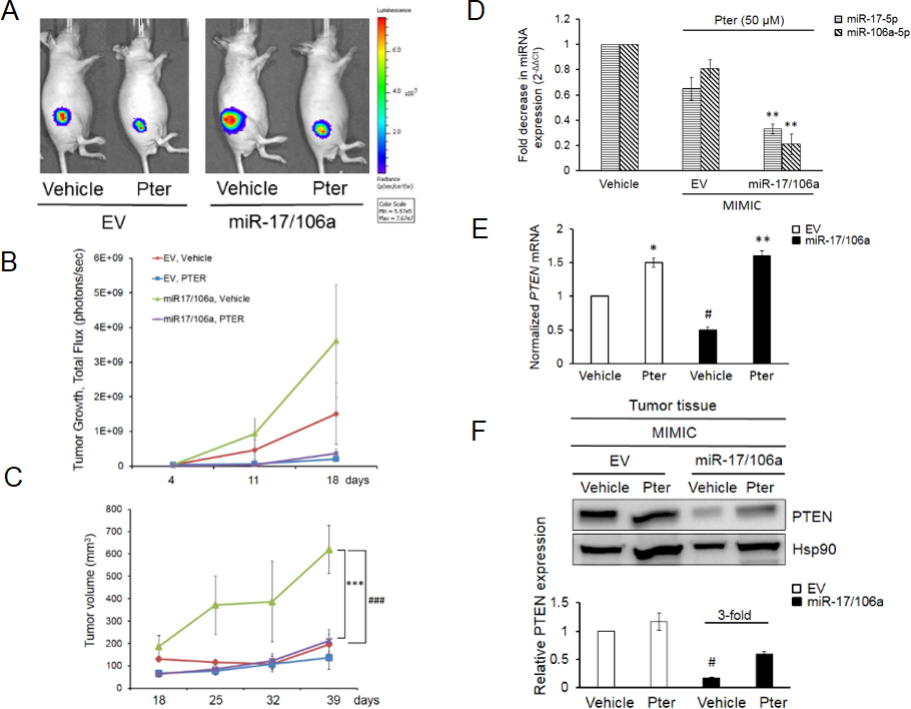
Pterostilbene diminishes the miR-17/106a-promoted tumor growth in prostate cancer xenografts **A.** Bioluminescent images of representative mice from each group (*n* = 8 mice per group) at day 39 are shown. **B.** Quantitative analysis of BL signals from day 4 after cancer cell injections until day 18 is shown. Light emission data in Total Flux (photons/sec) are plotted. **C.** Pterostilbene treatment considerably reduced tumor volumes in miR-overexpressing xenografts, which exhibited significantly accelerated tumor growth compared to EV control. Tumor growth was monitored using a digital caliper once a week starting at day 18. **D.** Pterostilbene down-regulated miR expression in 17/106a MIMIC tumors, measured by real time PCR. **E, F.** Pterostilbene rescued PTEN mRNA (E) and protein (F) expression in miR-17/106a MIMIC tumor tissues. Hsp90 was used as loading control and quantitation was performed using Image J software. Comparisons between EV and miR-overexpressing tumors (#) and vehicle and Pter-treated samples (*) are shown. #*p* < 0.05; ###*p* < 0.001; *p < 0.05; **p < 0.01; ***p < 0.001. Pter, pterostilbene.

**Figure 5:**
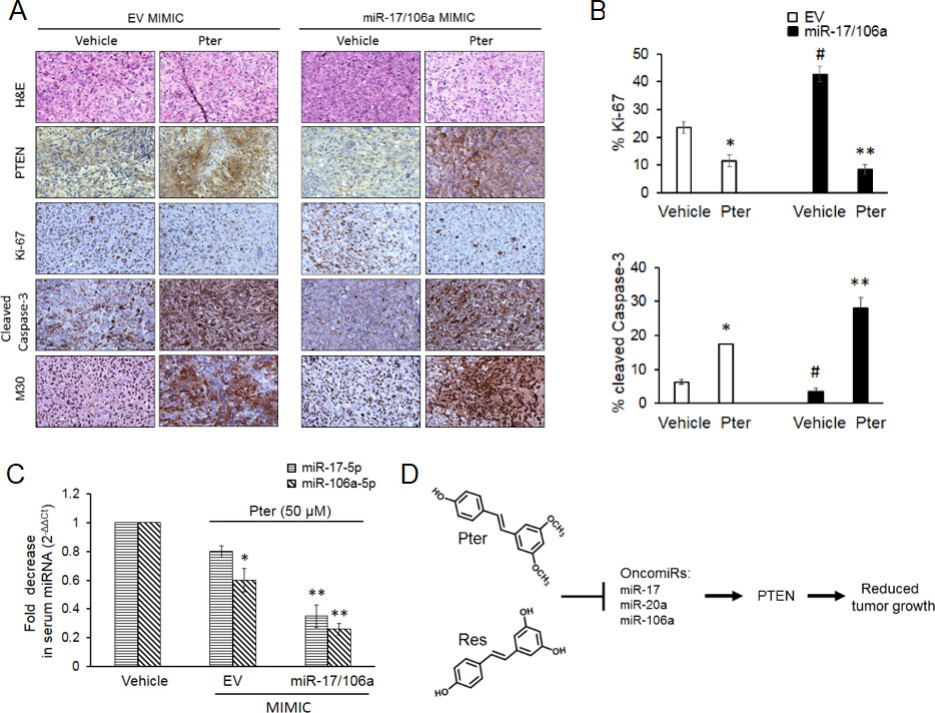
Pterostilbene induces PTEN expression and apoptosis, inhibits tumor cell proliferation and downregulates circulating tumor-derived oncomiRs *in vivo* **A.** Representative H&E and IHC images of PTEN, Ki-67 (proliferation); cleaved caspase-3 and M30 (apoptosis) staining in EV and miR-17/106a MIMIC xenografts upon pterostilbene treatment (magnification x100). **B.** Percent quantitation of Ki-67 and cleaved Caspase-3 staining is shown (*n* = 3 per group). **C.** Pterostilbene downregulated oncomiRs in serum of xenograft mice. Quantitative RT-PCR analysis of circulating levels of miRs-17-5p and -106a-5p in sera (*n* = 3 per group) from EV MIMIC and miR-17/106a MIMIC xenograft mice. Data represents the mean ± SEM of three independent experiments. Comparisons between EV and miR-overexpressing tumors (#) and vehicle and Pter-treated samples (*) is depicted. #*p* < 0.05; *p < 0.05; **p < 0.01. **D.** Proposed model of miR-regulated anticancer activity of dietary stilbenes. Pterostilbene and resveratrol inhibit expression of PTEN targeting miR-17 family oncomiRs, which leads to rescue of tumor suppressor PTEN expression resulting in inhibition of tumor growth. Pter, pterostilbene.

Meta-analysis for correlation of miR-17 and -106a expression in prostate cancer samples [37] clearly indicated a significant overexpression of these miRs in prostate cancer patients compared to normal cohort (Supplementary Figure S3) indicating their clinical significance in PCa and therefore the therapeutic value of targeting these miRNAs. In preclinical studies, to examine the miR-mediated anticancer efficacy of pterostilbene *in vivo*, we implanted DU145-Luc EV and miR-17/106a overexpressing cells on the right flank of male nude mice and treated them with vehicle (10% DMSO) or pterostilbene (50 mg/kg bw). We chose pterostilbene based on its better effects on miR downregulation leading to PTEN rescue (Figure 1A, Figure 3C, 3D) and its known potent pharmacokinetics including superior bioavailability and more effective tissue distribution over resveratrol [22, 30, 38–42]. Tumor growth was monitored weekly by both bioluminescent imaging and caliper measurements, which complemented each other, until day 39 when mice were sacrificed and tumors and sera collected for analysis (Figure 4A). Tumor measurements by bioluminescent imaging in the first 18 days clearly revealed that ectopic expression of miR-17/106a promotes tumorigenic properties of cells as evident by accelerated tumor progression and larger tumor volumes in miR-17/106a overexpressing xenografts as compared to the EV group (Figure 4B). Although still not reaching statistical significance at that time, miR-17/106a vehicle-treated tumors exhibited steady growth but revealed non-consistent measurements due to limitations of photon emission detection of large s.c. tumors that consist of necrotic areas and infiltrating host cells [31, 43–45]. On the other hand, caliper-based measurements were feasible only after day 18, after which they were more reliable than bioluminescent measurements and demonstrated statistically significant differences between miR-overexpressing tumors and EV by day 39 (Figure 4C). Importantly, pterostilbene treatment significantly decelerated tumor growth in miR-17/106a overexpressing xenografts without any obvious adverse effects on mice (Supplementary Figure S4A). Pterostilbene treatment downregulated miRs-17 and 106a more efficiently in miR-17/106a overexpressing tumors (Figure 4D) while simultaneously enhancing PTEN mRNA (Figure 4E) and protein expression by approximately three fold (Figure 4F). Immunohistochemical analysis revealed a decrease in PTEN expression along with a five-fold increase in Ki-67 and a three-fold decrease in cleaved Caspase-3 staining in miR-overexpressed tumors compared to that in controls (Figure 5A and 5B). Pterostilbene treatment in both groups, on the other hand, reversed these effects and demonstrated more potent effects in miR-overexpressing tumors (Figure 5A and 5B). Collectively, these data demonstrate that pterostilbene could target overexpressed miR levels and still restore PTEN expression leading to a decrease in tumor growth.

A number of studies have assessed the potential of circulating miRNAs to diagnose early-stage prostate cancer [7–9]. The ability of circulating miRNAs as predictors to treatment response or as chemopreventive markers is understudied. We questioned whether pterostilbene can diminish the abundance of secreted miRNAs and whether detection of changes in circulating miRs in response to treatment may be useful in the future clinical applications. To this end, we analyzed the relative abundance of circulating miR-17 and -106a in the sera from both groups of xenografts treated with pterostilbene. First of all, miR-17-5p and miR-106a-5p were highly expressed in sera collected from mice that were injected with miR-17/106a MIMIC cells compared to those that were carrying EV MIMIC cells (Supplementary Figure S4B). The results shown in Figure 5C indicated that in response to pterostilbene treatment reduction in circulating miR-17-5p and miR-106a-5p were more prominent in sera from ectopic miR-expressing xenografts compared to their control counterparts. This implied that beneficial anticancer effects of pterostilbene may be reflected through changes in levels of circulating miRs detected in serum.

## DISCUSSION

From a therapeutic perspective, dietary agents are being intensively studied for their anticancer properties. Nutriepigenomic studies, which are in their infancy, are focusing on bioactive dietary factors-induced beneficial changes in gene expression through epigenetic modifications [46]. Several studies have recently highlighted the anti-cancer role of dietary compounds via their effect on microRNAs aberrantly expressed in malignant cells [18, 19, 47]. An initial large scale miRnome analysis of lung, breast, stomach, prostate, colon and pancreatic tumors revealed high expression of miR-17 and miR -20a among others [2]. Among these oncomiRs, miR-17∼92 cluster, described as oncomiR 1 has been shown to be overexpressed in several cancers [48]. We detected significantly increased expression of miRs-17, -20a, -106a and -106b and the ability of dietary resveratrol to downregulate these oncomiRs in miRNA profiling studies of prostate cancer cells [15].

Our current work expands on these initial findings by demonstrating direct association between oncomiRs and their target, functional involvement of miRNAs in tumor manifestation, and, importantly, the ability of resveratrol and its potent analog pterostilbene to reverse silencing effects of miRNAs on tumor suppressor PTEN.

Although oncogenic potential of miR-17∼92 and miR-106b∼25 clusters in targeting 3′UTR of PTEN mRNA has been recently reported [13, 48], we investigated the ability of resveratrol and pterostilbene to counteract oncomiRs. Indeed, while ectopic overexpression of miRs -17, -20a and -106a induced a significant loss in PTEN 3′UTR luciferase activity with miR-17 revealing most striking down-regulation, both resveratrol and pterostilbene rescued this loss of luciferase signal in a dose-dependent manner, indicating that there is a direct effect of treatment on ectopically overexpressed miRNAs. As expected, the inhibitory effect of resveratrol and pterostilbene treatment on these oncomiRs was reflected in the elevated PTEN mRNA and protein expression levels.

Of note, resveratrol diminished the abundance of ectopically overexpressed oncomiRs in transiently transfected DU145 cells almost with the same potency as it did the endogenous expression of miRs-17 and -20a, indicating that there could be a possible affinity of resveratrol for a pool of oncomiRs, which are overexpressed in a system such as in transformed cells. This hypothesis is attested by our findings of relatively higher abundance of these oncomiRs and their better targeting by resveratrol in DU145 prostate cancer cells compared to “normal” RWPE-1 cells (data not shown). Since commercially available synthetic miRNAs are described as pre-miRs, which after introduction in the cellular system undergo processing by Dicer to generate mature miRNAs, it is plausible that resveratrol acts either directly on mature miRNAs and/or on the molecular substrates responsible for generating the mature miRNAs. There are evidence in literature for both possibilities. Hagiwara *et al*., have shown that resveratrol and more so pterostilbene enhances the transcriptional activation of certain tumor suppressive miRNAs and Argonaute-2 (Ago-2), a key regulator of miRNA homeostasis and biogenesis [49]. On the other hand, direct interaction of resveratrol with mature miRNAs, which could potentially alter their interaction with the target mRNA, was reported recently by Baselga-Escudero and colleagues [50]. No data is available on regulation of Dicer, Drosha or other miRNA machinery-related genes by dietary compounds in prostate cancer. We believe that there is a possibility for stilbenes to regulate other microRNA processing components including Dicer, which have been shown to be upregulated in prostate cancer [10, 51], subsequently diminishing cellular pools of oncomiRs. However, based on the differential effect of resveratrol on the expression of oncomiRs (downregulation) and tumor suppressor miRNAs (upregulation), it would be safe to assume that the specificity of the resveratrol-mediated downregulation of miR-17 family could come from resveratrol's regulation of some transcription factor(s) that regulate the transcription of these miRNA genes (unpublished data).

The more potent anticancer properties of pterostilbene can be explained by its chemical structure differences from resveratrol where two hydroxyl groups of resveratrol are replaced by two methoxy groups, which makes pterostilbene more stable, lipophilic and membrane-permeable [52]. We and others have reported earlier a more effective inhibition of prostate tumor cell growth *in vitro* and *in vivo* by pterostilbene, and essentially, greater accumulation of pterostilbene in serum and tissues compared to resveratrol [30, 38–41].

Proof-of concept *in vivo* studies using miR-17/106a overexpressing tumor cells clearly showed accelerated tumor development in immunocompromised mice that was efficiently counteracted upon pterostilbene treatment, which resulted in enhanced PTEN expression and apoptosis. This provides substantial preclinical evidence for the therapeutic ability of dietary stilbenes in regulating tumor development and progression via miR-mediated mechanisms.

Finally, to further potentiate the importance of our finding for relevance to clinical application, we determined the changes in circulating levels of miRs-17 and -106a from sera of tumor bearing mice. We found a three-fold higher expression of these tumor-derived oncomiRs in the sera of miR-overexpressing mice compared to their empty vector counterparts, which was efficiently downregulated by pterostilbene. Detection of serum circulating miRNAs in prostate cancer animal models and clinical cohorts of prostate cancer patients has been reported [7–9, 53], however, the regulation of circulating miRs in serum by dietary compounds has not been investigated. In this report, we demonstrated that pterostilbene treatment not only leads to reduction of tumor growth but also affects the levels of circulating miRs, which can be utilized as chemopreventive and predictive markers in prostate cancer.

In conclusion, we show here that high miRs-17, -20a, -106a and -106b expression is strongly correlated with prostate cancer progression, and confirm PTEN tumor suppressor as a key target of these oncomiRs in prostate cancer cells. Importantly, we demonstrate oncomiR-mediated anticancer effects of resveratrol and pterostilbene, which suggest that restoring PTEN expression may be a feasible approach for prostate cancer chemoprevention and therapy (Figure 5D). To the best of our knowledge, our study is the first to address the functional consequences of onco-miRNA downregulation by dietary pterostilbene *in vivo*. Also, for the first time, we demonstrate the modulation of circulating oncomiRs in murine serum in response to treatment by natural agent, which opens possibilities for miRNA utilization as chemopreventive biomarkers in prostate cancer. These findings provide a novel perspective on how dietary stilbenes may function as chemopreventive and anticancer agents by modulating miRNAs, alterations of which can be responsible for prostate cancer predisposition and progression.

## MATERIALS AND METHODS

### Reagents

Resveratrol was purchased from Sigma-Aldrich, Indianapolis, USA. Pterostilbene was synthesized following previously published procedures [54]. The purity of pterostilbene was determined to be >99%. Stock solutions of resveratrol and pterostilbene were made using high purity DMSO (MP Biomedicals, Solon, USA) and kept at 4°C, in the dark.

### Cell culture

Prostate cancer cell lines DU145 and 22Rv1 were purchased from ATCC, Manassas, USA and maintained in RPMI-1640 (Life Technologies, NY, USA) containing 10% FBS as described previously [28, 29]. Cells were maintained in an incubator at 37°C with 5% CO_2_. Both cell lines were last authenticated using short tandem repeat profiling at Research Technology Support Facility, Michigan State University in July 2014. Resveratrol and pterostilbene treatment were carried out as previously described [28–31].

### Cloning and dual- luciferase reporter assay

We amplified a 535 bp region of the *PTEN* 3′UTR encompassing the miR-17, -20a and -106b seed sequence and cloned into the Sac I (New England Biolabs, Ipswich, USA) site of pMIRGLO vector (Promega, Madison, USA) downstream of the Firefly luciferase gene using the InFusion HD cloning kit (Clontech, Mountain View, USA). The orientation of the insert was verified by sequencing (Davis Sequencing). To introduce three-point mutations into the seed sequence of the miRs binding site, we used the InFusion mutagenesis method (Clontech, Mountain View, USA) as per manufacturer's instructions. The wild type primers used were as follows: forward, 5′-CTA GTT GTT TAA ACG AGC TCT CTG ACA CCA CTG ACT CTG ATC CA-3′; reverse, 5′-GAC TCG AGG CTA GCG AGC TCA GTA GGC TTT GAA GGA CAG CAG GA-3′ (bold letters represent Sac I cloning site). The mutated primers used were as follows: forward, 5′-GGA TTA ATA AAG ATG GGA GTA TCC CG-3′; reverse, 5′-TTT CTG AGC ATT CCC TCC ATT CCC-3′ (underlined indicates mutated nucleotides). Luciferase reporter assay was performed using the Dual-Luciferase Reporter Assay System (Promega, Madison, USA). After cells were co-transfected with either wild type or mutant 3′UTR *PTEN* (800 ng) and 50 nM synthetic Pre-miRs -17 or -20a or -106b or with non-targeting miR Negative #1 (miR-NC, negative control, Life Technologies, NY, USA) treated with resveratrol/pterostilbene, luciferase activity was measured on a Synergy 4 plate reader (Biotek Instruments). Firefly luciferase activity was normalized for transfection efficiency to Renilla luciferase activity. The ratio of *PTEN* 3′UTR alone to Empty Vector (EV) alone (3′UTR/EV) was set as control (Ctrl = 1) and all changes in luciferase activity were calculated relative to Ctrl.

### Generation of DU145 luciferase expressing cells

DU145 luciferase (DU145-Luc) cells were established, expanded and tested for their Luc activities using an IVIS Spectrum (Perkin Elmer, Waltham, USA) as described previously [30].

### Stable overexpression of miR-17/106a in DU145-Luc cells

DU145-Luc cells were stably transduced with commercially available miRIDIAN shMIMIC lentiviral microRNA particles expressing miR-17/106a (hsa-miR-106a miRIDIAN shMIMIC), termed as miR-17/106a MIMIC and Empty shMIMIC lentiviral particles, termed as EV MIMIC (Thermo Fisher Scientific, Waltham, USA). MiR-17 and miR-106a share the same seed sequence except for a cytosine residue in miR-17replaced by an adenine in miR-106a at the 5′ end. The plasmid is based on the pSMART vector with a puromycin selection and GFP cassette. Cells were transduced using OptiMEM medium (Life Technologies, NY, USA) which contained 6 μg/ml polybrene (Sigma-Aldrich, Indianapolis, USA) with lentiviral particles at multiplicity of infection (MOI) = 8. On day 4 post-transduction, puromycin (Sigma-Aldrich, Indianapolis, USA) selection was initiated and GFP-positive clones were selected using cloning cylinders. Cells were plated in 24-well plates for expansion and propagated at a final concentration of 200 μg/ml puromycin.

### Quantitative real-time RT-PCR

RNA was isolated from DU145 and 22Rv1 cells using miRNeasy Mini Kit (Qiagen) as previously described [15]. Real-time PCR was performed using custom primers specific for hsa-miR-17-5p, hsa-miR-20a, hsa-miR106a-5p, hsa-miR-106b and miRCURY LNA™ Universal RT microRNA PCR kit (Exiqon, Vedbaek, Denmark). SNORD44 was used an internal reference. Specific PrimeTime™ human PTEN primers were used: forward 5′-GAACTTGTCTTCCCGTCGT-3′ and reverse 5′-AATGTTCAGTGGCGGAACT-3′ (Integrated DNA Technologies, Coralville, USA). For estimation of tumor-derived circulating miRNAs in murine serum, miRNA isolation was performed with 50 μl of sera. Each sample was spiked with 5 nM of Cel-miR-39 (Integrated DNA Technologies, Coralville, USA) from *Caenorhabditis elegans* as an internal reference control (Exiqon, Vedbaek, Denmark). Fold changes in miRNA/mRNA expression was estimated by the 2^−ΔΔCt^ method [55].

### Western blot

Whole-cell lysis was performed as previously described [15, 27]. The membranes were probed with anti-PTEN (1:1000, Cell Signaling, Danvers, USA) and anti-β-actin antibodies (1:2500, Santa Cruz Biotechnologies, Dallas, USA). Signals were visualized using the SuperSignal West Dura chemiluminescence kit (Thermo Fisher Scientific, Waltham, USA) and imaged on a Chemidoc gel imager (Bio-Rad, Hercules, USA). Images were quantified using the Image J software (NIH).

### Cell viability assay

Proliferation of cells treated with resveratrol and pterostilbene was determined by MTT (Sigma-Aldrich, Indianapolis, USA) assay as described [56]. Absorbance of the formazan was measured using Synergy-4 plate reader after 72 h of treatment. IC_50_ values were calculated by the linear interpolation method using MS Excel.

### Prostate cancer xenografts

Fox n1^nu/nu^ male mice were purchased from Harlan Laboratory at seven weeks of age. Housing and care of all animals were in accordance with the guidelines established by the University's Institutional Animal Care and Use Committee. Upon arrival, all animals were given a phytoestrogen-free AIN-76A diet (Research Diets, New Brunswick, USA) *ad libitum*. Mice were randomly divided into two groups of 16 mice each for both cells lines. 2 × 10^6^ DU145-Luc EV MIMIC or miR-17/106a MIMIC cells in combination with 50% matrigel (BD Biosciences, San Jose, USA, total volume 200 μl) were inoculated subcutaneously (s.c.) on the dorsal right flank of mice. On day 4, bioluminescence was measured in all animals and based on the total flux signal intensities, mice in each group were randomized into two subgroups each (*n* = 8): vehicle (10% DMSO) and treated with pterostilbene (50 mg/kg bw). Intraperitoneal injections (i.p.) were performed daily, 5 days a week, for 39 days. Bioluminescent and caliper measurements were taken weekly as previously described [29–31]. Calculation of tumor volume by digital Vernier caliper measurements were done by using formula as before [29, 31]. The mice were sacrificed at day 39 and tumors were excised. Total RNA and protein were isolated and the remaining tissue was placed in 10% formalin for histological and immunohistochemical (IHC) analysis. Blood was also collected at sacrifice, and serum samples were stored at −80°C until further analysis.

### Immunohistochemistry

Four μm thick formalin-fixed paraffin embedded tumor sections were stained as per the protocol described previously [30] to evaluate PTEN (1:250, Cell Signaling, Danvers, USA), Ki-67 (1:100, Abcam, Cambridge, USA), cleaved-Caspase 3 (1:500, Cell Signaling, Danvers, USA) and M30 (1:100, Roche, Indianapolis, USA). The VECTASTAIN ABC Elite kit and the ImmPACT DAB kit (Vector Laboratories, Burlingame, USA) were used to visualize staining. Images were recorded on a Nikon Eclipse 80i microscope. Ki-67 and cleaved-Caspase-3 stained nuclei were quantitated using ImageTool software.

### GEO database analysis

The data related to GSE21036 were acquired from Gene Expression Omnibus (GEO) website. We used the Bioconductor and GEO query package to access the expression data, platform and clinical data of the study. The expression data were log2-transformed and processed by the between-array variance stabilization normalization. The expression levels of given microRNA were summarized by boxplot in the tumor and normal sample subgroups separately.

### Statistical analysis

The differences between groups were analyzed by the two-sample two-tailed Student's *t* test. For animal studies, one-way ANOVA was used to assess the difference in mean among given groups. Levene's test was performed to test equal-variance assumption. The Welch's one-way ANOVA method was used when unequal variances were present. The pairwise comparison was conducted when significant differences in mean were verified. Bonferroni adjusted *p* values were reported in pairwise comparisons. Statistical significance was set as *p* ≤ 0.05. All data are cumulative of at least three independent experiments.

## SUPPLEMENTARY FIGURES


