# Supplementary material for: Resveratrol and pterostilbene epigenetically restore PTEN expression by targeting oncomiRs of the miR-17 family in prostate cancer
Source: Oncotarget. 2015 Aug 6;6(29):27214–26. doi: 10.18632/oncotarget.4877 (PMC4694984; doi:10.18632/oncotarget.4877)
Supplement: Supplementary file 1 [file oncotarget-06-27214-s001.pdf]

SUPPLEMENTARY FIGURES

"Resveratrol and pterostilbene epigenetically restore PTEN expression by targeting oncomiRs of the miR-17 family in prostate cancer"

3'UTR of human *PTEN* mRNA

...ATTTTTTTTTATCAAGAGGGATAAAACACCA
TGAAAATAAACTTGAATAAACTGAAAATGGAC
CTTTTTTTTTTTAATGGCAATAGGACATTGTG
TCAGATTACCAGTTATAGGAACAATTCTCTTT
TCCTGACCAATCTTGTTTTACCCTATACATCC
ACAGGGTTTTGACACTTGTTGTCCAGTTGAAA
AAAGGTTGTGTAGCTGTGTCATGTATATACCT
TTTTGTGTCAAAAGGACATTTAAAATTCAATT
AGGATTAATAAAGATGGCACTTTCCCGTTTTA
TTCCAGTTTTATAAAAAGTGGAGACAGACTGA
TGTGTATACGTAGGAATTTTTTCCTTTTGTGT
TCTGTCACCAACTGAAGTGGCTAAAGAGCTTT
GTGATATACTGGTTCACATCCTACCCCTTTGC
ACTTGTGGCAACAGATAAGTTTGCAGTTGGCT
AAGAGAGGTTTCCGAAGGGTTTTGCTACATTC
TAATGCATGTATTCGGGTTAGGGGAATGGAGG
GAATGCTCAGAAAGGAAATAATTTTATGCTGG
ACTCTGGACCATATACC...

Supplementary Figure S1: Sequence of human PTEN 3' UTR (535 bp) cloned in pMIRGLO vector. The seed match (GCACTTT, position 272) for miR-17, -20a and -106b is underlined.

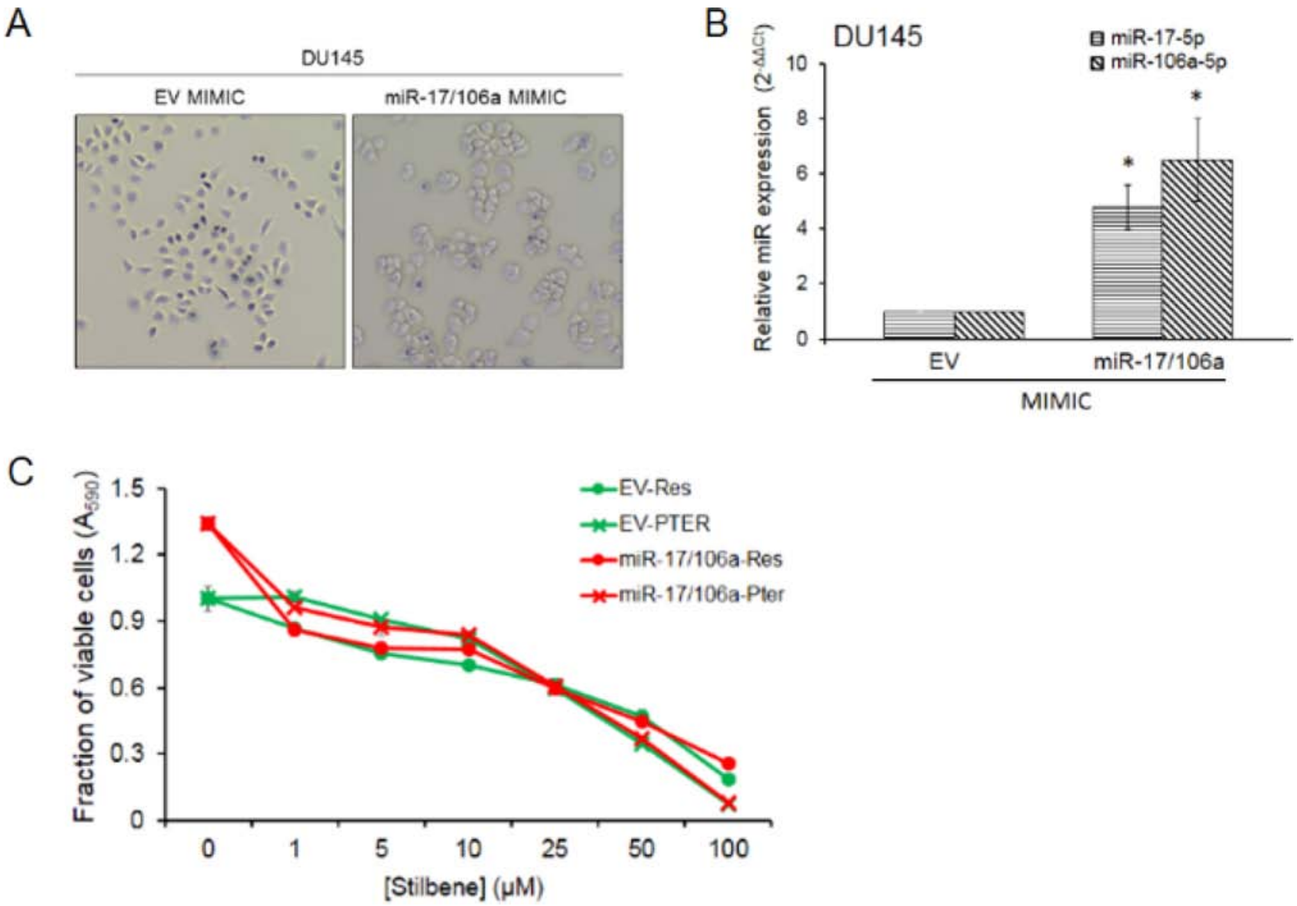

Supplementary Figure S2: Characterization of miR-17/106a overexpressing DU145-luciferase cells. A. Stable transduction of miR-17/106a MIMIC in DU145-luciferase cells showed altered morphology compared to EV MIMIC cells. The cells transduced with miR-17/106a appeared relatively rounded and enlarged compared to cells transduced with EV MIMIC, which assumed regular triangle-shaped appearance of DU145 cells (Magnification x40). **B.** Relative expression of miRs-17-5p and 106a-5p in stably transfected cells. **C.** Comparison of Res and Pter treatment on DU145 EV and DU145- miR-17/106a MIMIC cell viability *in vitro*. Data represent the mean ± SEM from three independent experiments. IC50 was calculated for Res (28.2 vs 33.7) and Pter (11.1 vs 11.3) in EV and miR-17/106a cells, respectively.

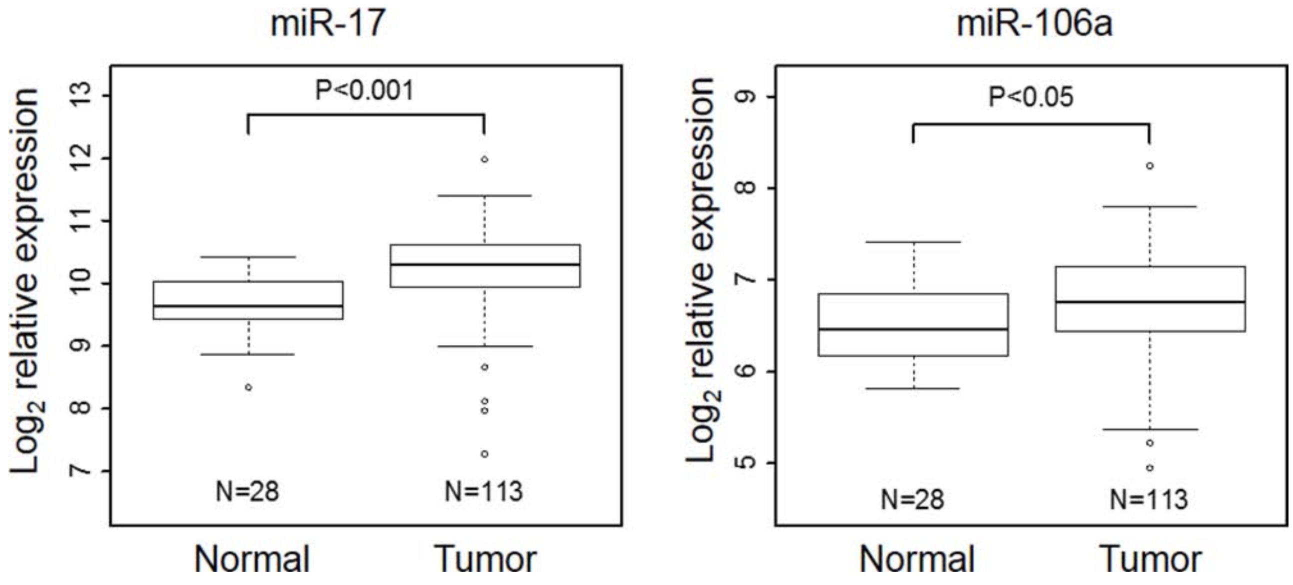

Supplementary Figure S3: Significant overexpression of miR-17 and miR-106a in human prostate cancer samples. The expression data acquired from Gene Expression Omnibus (GEO) related to GSE21036 was log2-transformed and represented. Comparison between normal and tumor samples was evaluated by the two-sample *t* test.

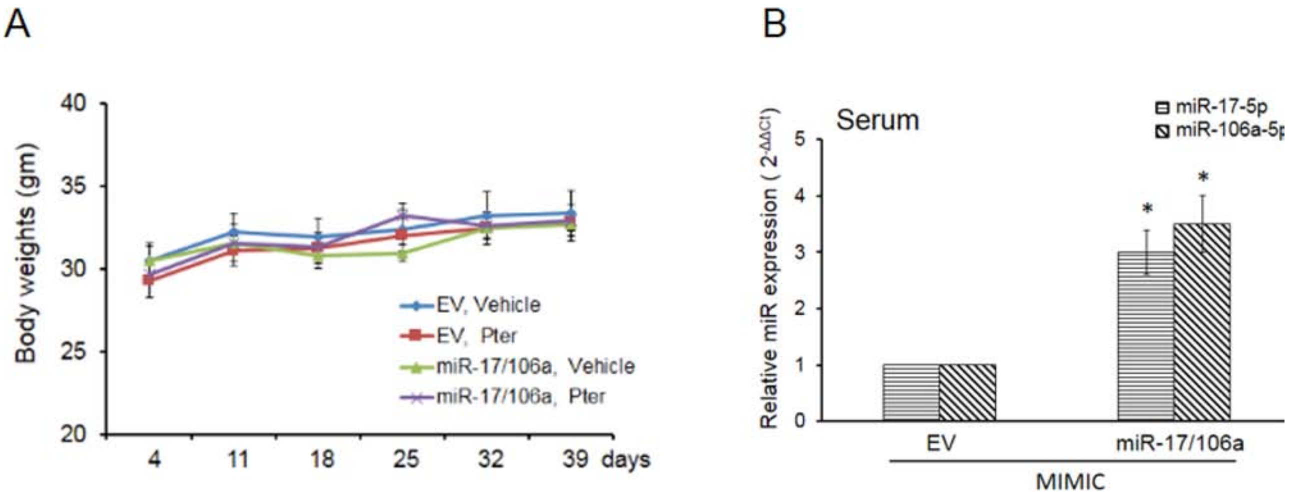

Supplementary Figure S4: A. Body weight analysis of nude mice with EV MIMIC and miR-17/106a MIMIC tumors in vehicle and pterostilbene treated groups (n = 8 mice per group). Mice were weighed once per week for 6 weeks. There were no significant differences in the body weights of mice among all groups ($p = 0.61$). **B.** Relative expression of miR-17-5p and miR106a-5p in serum samples from EV and miR-overexpressing xenografts. Data represents the mean ± SEM from two independent experiments (n = 3 mice per group), *$p < 0.05$.
